# Supplementary material for: BMSC-derived exosomal miR-27a-3p and miR-196b-5p regulate bone remodeling in ovariectomized rats
Source: PeerJ. 2022 Sep 22;10:e13744. doi: 10.7717/peerj.13744 (PMC9509671; doi:10.7717/peerj.13744)
Supplement: Table S1 — ACP5, tartrate resistant acidic phosphatase; CTsK, cathepsin K; NFATc1, nuclear factor of activated T-cell cytoplasmic 1; ALP, alkaline phosphatase; OCN, osteocalcin; OSX, osterix; RUNX2, runt-related transcription factor 2. [file peerj-10-13744-s001.doc]

**Table S1.** Primer sequences used for real-time quantitative reverse transcription-PCR

| *Gene Name* | *Forward Primer (5’-3’)* | *Reverse Primer (5’-3’)* |
| --- | --- | --- |
| ACP5 | ATGACGCCAATGACAAGAGGTTCC | TTGTGCCGAGACATTGCCAAGG |
| CtsK | TGGCTGTGGAGGCGGCTATATG | CGGGTAAGCGTCTTCAGAGTCAA |
| NFATc1 | AGGCGTCCTCCTATGAGTCCAAC | CTCCTCTGGGTCCGTGGTCTTG |
| ALP | TCCATGGTGGATTATGCTCA | TTCTGTTCCTGCTCGAGGTT |
| OCN | GGAGGGCAGTAAGGTGGTGAA | GAAGCCAATGTGGTCCGCTA |
| OSX | GCCTACTTACCCGTCTGACTTTGC | CCCTCCAGTTGCCCACTATTGC |
| RUNX2 | GGACCGACACAGCCATATAAA | GCCTCATTCCCTAACCTGAAA |
| β-actin | GGCACAGTCAAGGCTGAGAATG | ATGGTGGTGAAGACGCCAGTA |
| miR-27a-3p | TTCACAGTGGCTAAGTTCCGC | Uni-Reverse Primer |
| miR-196b-5p | CGTAGGTAGTTTCCTGTTGTTGGG | Uni-Reverse Primer |
| cel-miR-39-3p | RiboBio, miRA0000010 | Uni-Reverse Primer |
| U6 | RiboBio, miRAN0002 | Uni-Reverse Primer |

ACP5, tartrate resistant acidic phosphatase; CTsK, cathepsin K; NFATc1, nuclear factor of activated T‐cell cytoplasmic 1; ALP, alkaline phosphatase; OCN, osteocalcin; OSX, osterix; RUNX2, runt-related transcription factor 2.
