# Supplementary material for: BMSC-derived exosomal miR-27a-3p and miR-196b-5p regulate bone remodeling in ovariectomized rats
Source: PeerJ. 2022 Sep 22;10:e13744. doi: 10.7717/peerj.13744 (PMC9509671; doi:10.7717/peerj.13744)

**Uncropped scans of Western blot images**

Fig. 2 (b) Western blot indicats the expression of CD81, Hsp70, TSG101 and Calnexin in cellular and exosomal lysates of BMSCs.


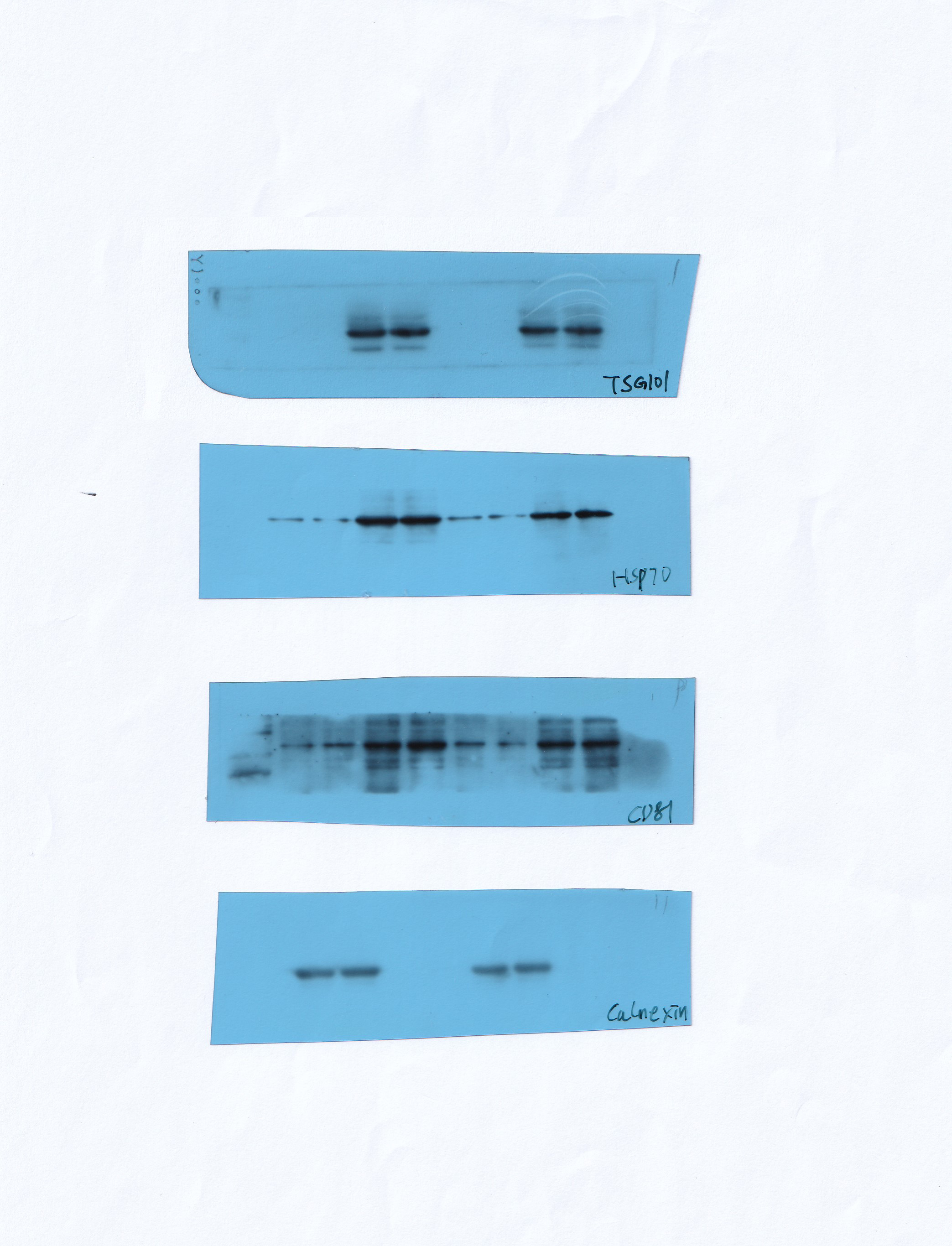

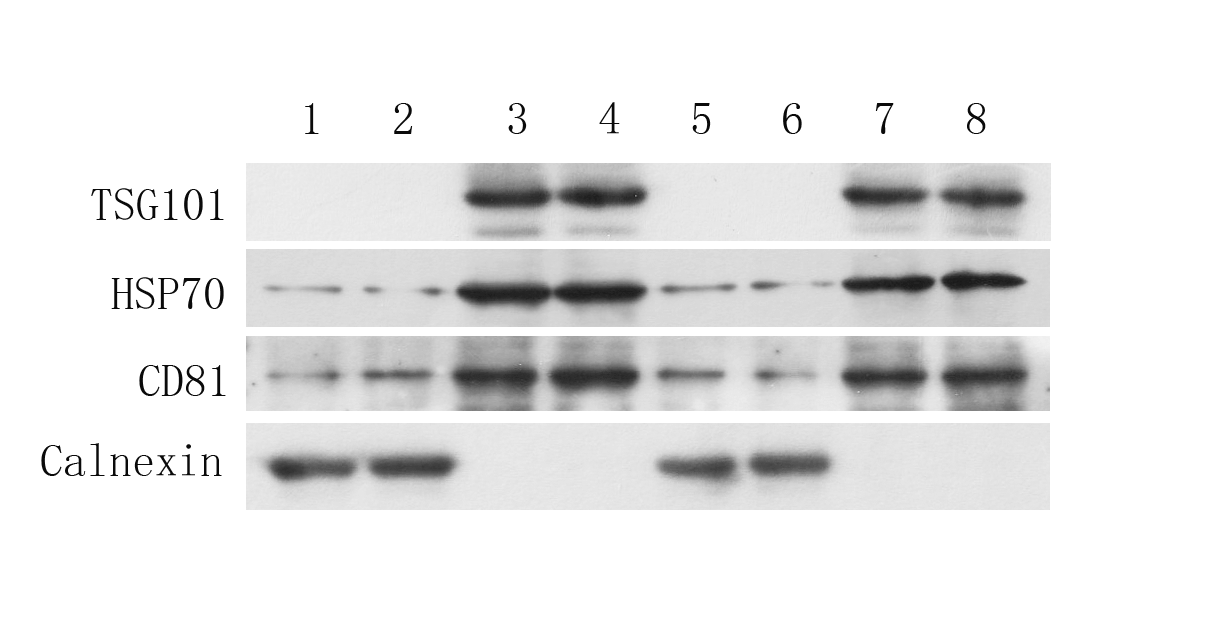


**Fig. 6** (d) The protein levels of ALP, OCN, OSX and RUNX2 in BMSCs transfected with miR-27a-3p mimic/inhibitor detected by western blot.


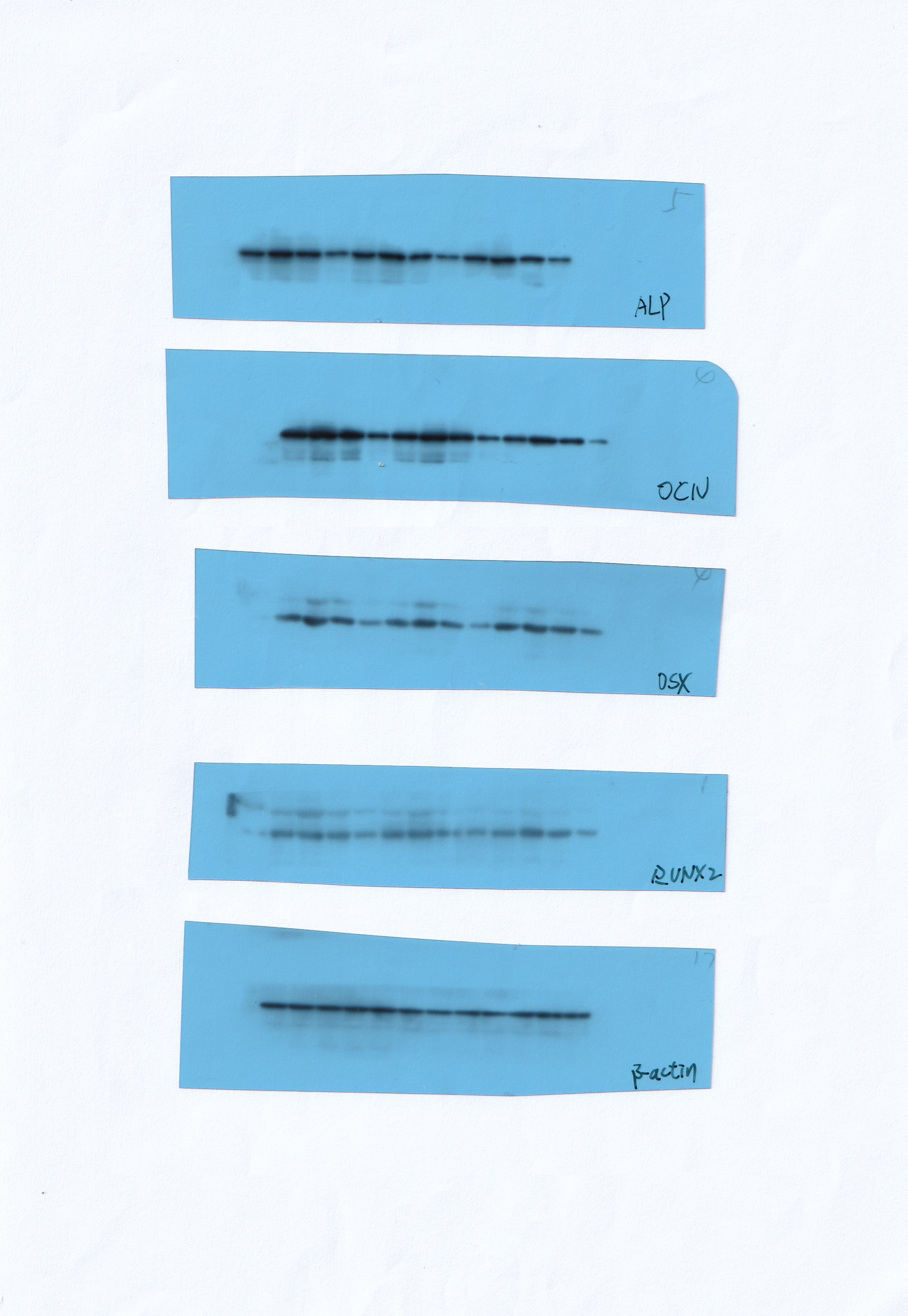

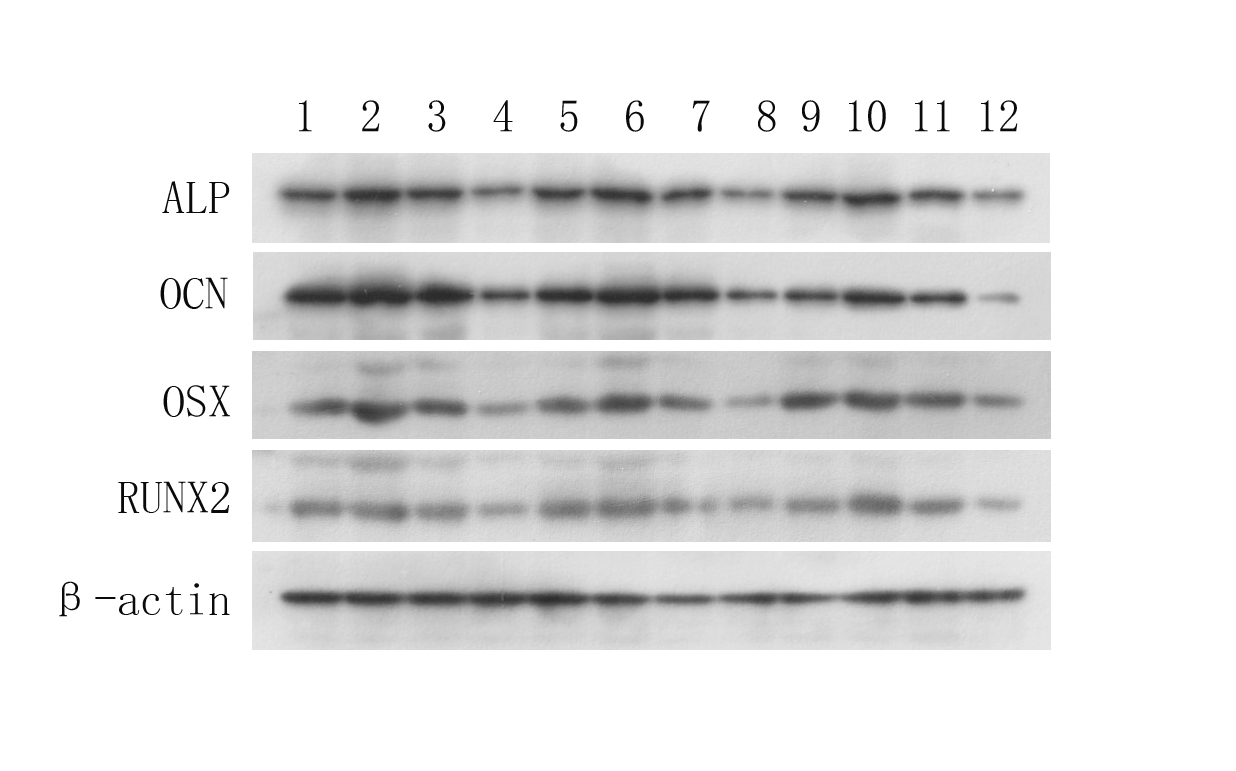


**Fig. 6** (j) The protein levels of ALP, OCN, OSX and RUNX2 in BMSCs transfected with miR-196b-5p mimic/inhibitor detected by western blot.


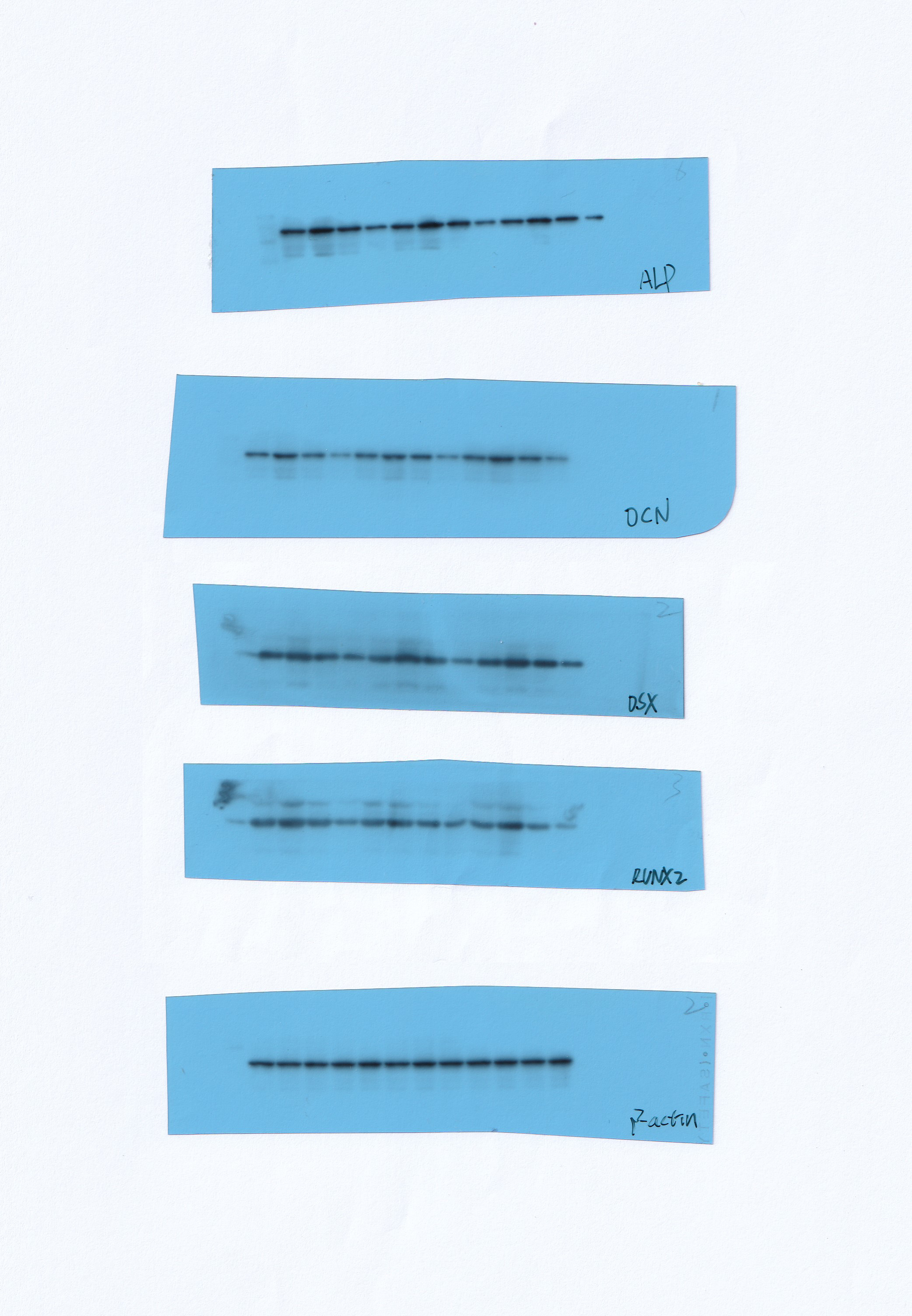

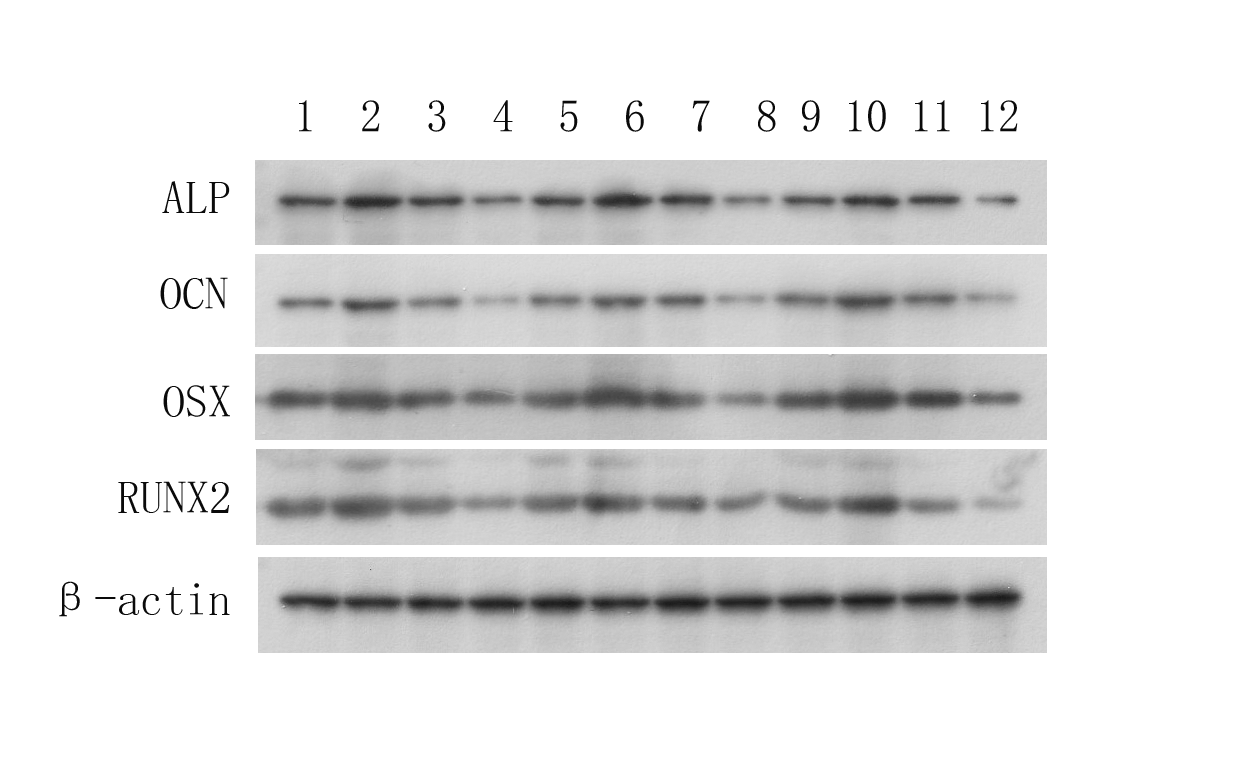

Supplement: Supplemental Information 1 [file peerj-10-13744-s006.doc]
